# Supplementary material for: The causal role of circulating inflammatory markers in osteoporosis: a bidirectional Mendelian randomized study
Source: Front Immunol. 2024 Jul 18;15:1412298. doi: 10.3389/fimmu.2024.1412298 (PMC11291241; doi:10.3389/fimmu.2024.1412298)
Supplement: Supplementary file 1 [file DataSheet_1.zip › supplementary material/CIM-OSTEOPOROSIS /GCST90274839.csv_forest.pdf]

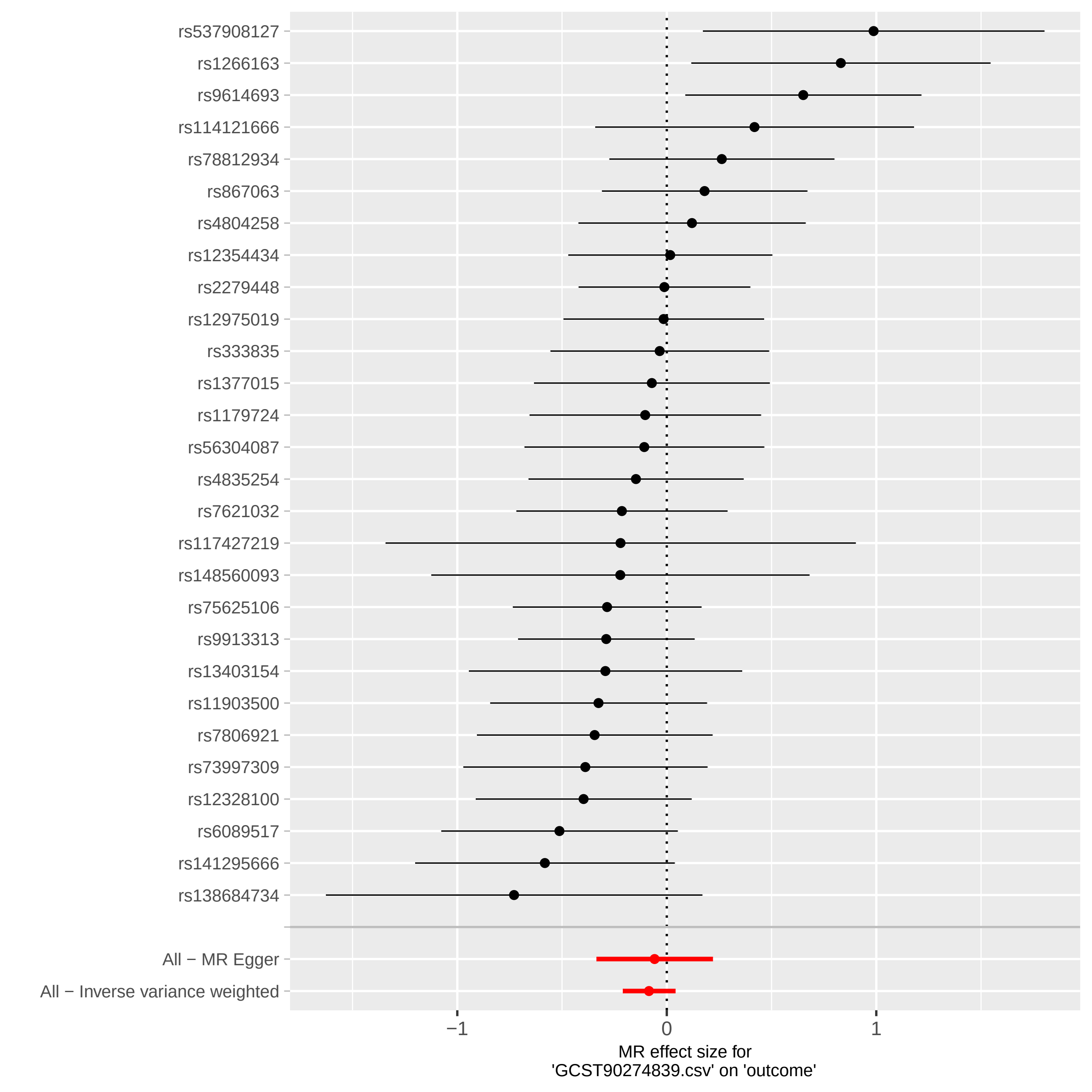
rs537908127
rs1266163
rs9614693
rs114121666
rs78812934
rs867063
rs4804258
rs12354434
rs2279448
rs12975019
rs333835
rs1377015
rs1179724
rs56304087
rs4835254
rs7621032
rs117427219
rs148560093
rs75625106
rs9913313
rs13403154
rs11903500
rs7806921
rs73997309
rs12328100
rs6089517
rs141295666
rs138684734
All − MR Egger
All − Inverse variance weighted
−1
0
1
MR effect size for
'GCST90274839.csv' on 'outcome'
